# Mathematical modelling supports the existence of a threshold hydrogen concentration and media-dependent yields in the growth of a reductive acetogen

**DOI:** 10.1007/s00449-020-02285-w

**Published:** 2020-01-25

**Authors:** Nick W. Smith, Paul R. Shorten, Eric Altermann, Nicole C. Roy, Warren C. McNabb

**Affiliations:** 1 School of Food and Advanced Technology, Massey University, Private Bag 11-222, Palmerston North, New Zealand; 2 Riddet Institute, Massey University, Private Bag 11222, Palmerston North, 4442 New Zealand; 3 AgResearch Ruakura Research Centre, Private Bag 3123, Hamilton, 3240 New Zealand; 4 AgResearch Grasslands Research Centre, Private Bag 11008, Palmerston North, 4442 New Zealand; 5 High-Value Nutrition National Science Challenge New Zealand, Auckland, New Zealand

**Keywords:** Mathematical modelling, Acetate, Hydrogen, Syngas, Threshold, Yeast extract

## Abstract

The bacterial production of acetate via reductive acetogenesis along the Wood–Ljungdahl metabolic pathway is an important source of this molecule in several environments, ranging from industrial bioreactors to the human gastrointestinal tract. Here, we contributed to the study of reductive acetogens by considering mathematical modelling techniques for the prediction of bacterial growth and acetate production. We found that the incorporation of a hydrogen uptake concentration threshold into the models improves their predictions and we calculated this threshold as 86.2 mM (95% confidence interval 6.1–132.6 mM). Monod kinetics and first-order kinetics models, with the inclusion of two candidate threshold terms or reversible Michaelis–Menten kinetics, were compared to experimental data and the optimal formulation for predicting both growth and metabolism was found. The models were then used to compare the efficacy of two growth media for acetogens. We found that the recently described general acetogen medium was superior to the DSMZ medium in terms of unbiased estimation of acetogen growth and investigated the contribution of yeast extract concentration to acetate production and bacterial growth in culture. The models and their predictions will be useful to those studying both industrially and environmentally relevant reductive acetogenesis and allow for straightforward adaptation to similar cases with different organisms.

## Introduction

The short-chain fatty acid acetate may be formed via a number of bacterial metabolic pathways, either from more complex organic compounds, or from the combination of two single carbon molecules. Reductive acetogenesis via the Wood–Ljungdahl metabolic pathway is an example of the latter, in which acetate may be formed from the combination of CO_2_ and/or CO with hydrogen [1]. Over 100 species of acetogenic bacteria that use this pathway have been isolated from a range of anaerobic habitats, such as the human and bovine gastrointestinal tract (GIT), oil fields and freshwater sediments [2].

In the human GIT, acetogens, alongside methanogens, may cross-feed on the hydrogen and CO_2_ produced by saccharolytic members of the GIT microbial population (for a review, see Smith et al. [3]). High concentrations of hydrogen in the GIT reduce the efficiency of carbohydrate breakdown by many microbes [4–6] and the presence of an acetogen has been shown to mitigate this effect in rodents [7]. Acetogens have also been investigated as a potential means of reducing the amount of hydrogen converted to the greenhouse gas methane in the rumen [8, 9], since they have proven effective hydrogen consumers in the GIT of other animals [10, 11].

Industrially, the use of acetogens to reduce waste and produce biofuels and other useful chemicals is an area of current development (for a review, see Bengelsdorf et al. [12]). Waste gases containing high concentrations of CO_2_ and CO can be combined with hydrogen to form synthesis gas (syngas) and then utilised as the substrate for reductive acetogenesis in bioreactors, resulting in the formation of acetate, ethanol and a number of other useful compounds [13].

A greater understanding of the growth and metabolic dynamics of acetogenic bacteria would be beneficial for several scientific fields, from human and animal nutrition and well-being, through to biotechnology and bioengineering. In this paper, we work towards an optimum method for modelling hydrogen consumption by acetogens. The established Monod model for microbial growth may not be sufficient to capture hydrogen uptake by these bacteria, due to the proposed existence of a minimum hydrogen concentration required for acetogen growth [14]. Therefore, we present a number of mathematical modelling techniques that include a substrate threshold and apply these models to experimental data for the acetogen *Blautia hydrogenotrophica* (originally named *Ruminococcus hydrogenotrophicus* [15]*,* but reclassified [16]). We used these models to investigate the growth of this bacterium on different media. *B. hydrogenotrophica* was chosen due to the availability of time-course data for its growth, hydrogen consumption and acetate production in monoculture. It is also present in the human GIT [15] and is potentially suitable for industrial applications [17].

## Materials and methods

### Assumptions

Our model considered only two of the metabolites involved in reductive acetogenesis: hydrogen and acetate (CH_3_COO–). All other molecules necessary for bacterial growth were assumed to be abundantly available in the medium throughout the experiment, including CO_2_.

We did not consider the influence of gas–liquid transfer in this model. Although the hydrogen and CO_2_ necessary for reductive acetogenesis were added to the gaseous phase, we assumed that mass transfer of these metabolites into the aqueous phase was not a limiting factor. Including mass transfer in the model would be challenging due to the lack of available data for the aqueous concentration of these molecules in the experiments considered. This assumption also reduced the complexity of the model and the number of parameters to be estimated. Experimental work has emphasised the need for aqueous metabolite data in the precise estimation of threshold concentrations under continuous metabolite inflow [18]. Under the batch conditions considered here, the estimate of threshold concentrations should be minimally influenced by mass transfer limitation. However, the absence of mass transfer in this model may result in underestimates of growth rates.

We assumed the following stoichiometry for reductive acetogenesis along the Wood–Ljungdahl pathway [1]:

$${\text{4H}}_{{2}} + {\text{ 2CO}}_{{2}} \to {\text{ Acetate }} + {\text{ H}}^{ + } + {\text{ 2H}}_{{2}} {\text{O}}$$

### Data capture

We obtained data from literature sources via image capturing in MATLAB (The MathWorks; www.mathworks.com). Where necessary, cell dry weight (CDW) in g L^−1^ was determined using the optical density (OD) conversion for *B. hydrogenotrophica* of CDW = 0.37 ⋅ OD [19]. Where metabolite data was given in g L^−1^, we used the conversion $x$ [mM] = $\frac{x [\mathrm{g} { \mathrm{L}}^{-1}] }{2.016}$⋅ 1000 for hydrogen and $x$ [mM] = $\frac{x [\mathrm{g} {\mathrm{L}}^{-1}]}{59.044}$⋅ 1000 for acetate.

### Model fitting

The model was calibrated using time-course growth and metabolite data from Bernalier et al. [15] and validated using data from Groher, Weuster–Botz [19]. The data was sampled using image capturing and graphical input software in MATLAB (The MathWorks; www.mathworks.com). To obtain estimates for parameter values and the reliability of these estimates, a Markov chain Monte Carlo (MCMC) method was employed, implemented in MATLAB. The optimisation objective function chosen was the minimisation of the sum of normalised squared differences between the model prediction and the experimental data. 10^6^ iterations of the MCMC algorithm were performed for each model structure. Goodness of fit was assessed from *R*^2^ values.

### Mathematical model

We chose Monod kinetics as the starting point for our model [20], with the inclusion of a constant cell death rate and the production of acetate, giving the following system of differential equations:

1 $$\frac{\mathrm{d}H}{\mathrm{d}t}=-\frac{{\mu }_{\mathrm{m}\mathrm{a}\mathrm{x}} X}{Y}\left(\frac{H}{{K}_{H}+H}\right),$$

2 $$\frac{\mathrm{d}P}{\mathrm{d}t}=-\frac{1}{4}\frac{\mathrm{d}H}{\mathrm{d}t},$$

3 $$\frac{\mathrm{d}X}{\mathrm{d}t}=-Y\frac{\mathrm{d}H}{\mathrm{d}t}-{k}_{d}X,$$

where $H$, $P$ and $X$ are the concentrations of the substrate hydrogen (mM), the product acetate (mM) and bacterial cells (g L^−1^ CDW) in the medium, respectively. Time ($t$) is measured in hours. ${\mu }_{\mathrm{m}\mathrm{a}\mathrm{x}}$ is the maximum growth rate of the bacterium (h^−1^) and $Y$ is the yield of bacterial cells per substrate consumed (g L^−1^ mM^−1^). ${K}_{H}$ is the half-saturation constant (mM) for hydrogen uptake by the bacterium. Finally, ${k}_{d}$ is the death rate (h^−1^), assumed to be constant throughout the experiment. We assumed that only a negligible proportion of substrate is used for cell maintenance.

The Monod kinetics model described above is widely applied to model microbial growth. However, it is known that explicitly identifying the values of ${\mu }_{\mathrm{m}\mathrm{a}\mathrm{x}}$ and ${K}_{H}$ is not always possible using model fitting, due to strong correlation between estimates of these two parameters obtained from a typical single time-course experiment [21]. In our modelling, we found that it was only possible to estimate the ratio of these two parameters; therefore we re-parameterised Eqs. 1–3 as follows:

4 $$\frac{\mathrm{d}H}{\mathrm{d}t}=-\frac{ \eta X H}{Y},$$

5 $$\frac{\mathrm{d}P}{\mathrm{d}t}=-\frac{1}{4}\frac{\mathrm{d}H}{\mathrm{d}t},$$

6 $$\frac{\mathrm{d}X}{\mathrm{d}t}=-Y\frac{\mathrm{d}H}{\mathrm{d}t}-{k}_{d}X .$$

**Fig. 1:**
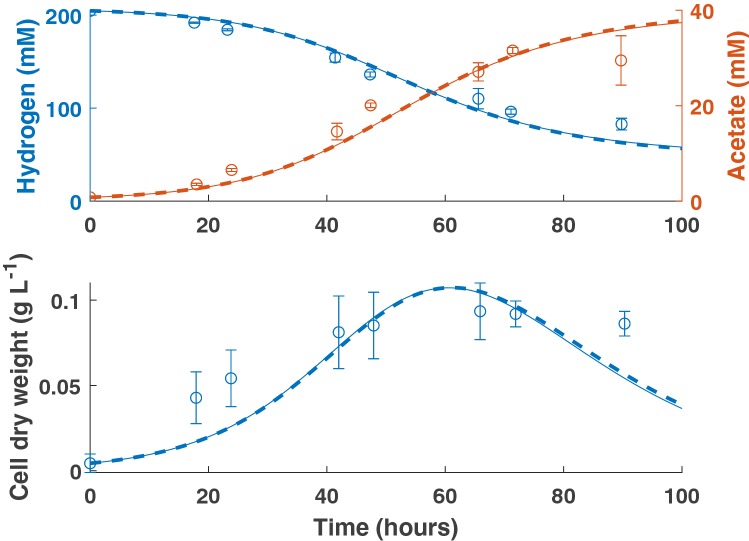
Comparison of Monod kinetics (solid line; Hydrogen *R*^2^ = 0.96; Acetate *R*^2^ = 0.94; CDW *R*^2^ = 0.76) with first-order kinetics (dashed line; Hydrogen *R*^2^ = 0.96; Acetate *R*^2^ = 0.94; CDW *R*^2^ = 0.76) when fit to monoculture data for *B. hydrogenotrophica*. Error bars denote standard deviations from three experimental determinations

Here, we have used first-order kinetics rather than Monod kinetics, where $\eta \simeq \frac{{\mu }_{\mathrm{m}\mathrm{a}\mathrm{x}}}{{K}_{H}}$ (h^−1^ mM^−1^; [22]). As shown in Fig. 1, there is negligible difference between the MCMC-generated best fit for the first-order kinetics model and the Monod model when applied to monoculture data for *B. hydrogenotrophica* from Bernalier et al. [15]. Linearization of the Monod model to first-order kinetics has been successfully applied elsewhere [22], and the need for it is shown by the poor reliability of the estimate of ${K}_{H}$ used in Fig. 1. We obtained a ${K}_{H}$ value of 37,328 mM from model fitting, with a 95% confidence interval of 1770–77,680 mM and there was no chain convergence of the MCMC. The correlation coefficient between ${\mu }_{\mathrm{m}\mathrm{a}\mathrm{x}}$ and ${K}_{H}$ was 0.93, emphasising the poor reliability of estimating these two parameters individually from the available data. The first-order kinetics model is therefore selected for further use due to more reliable parameter estimation with this model structure.

One aspect of the data that is challenging to capture with either Monod or first-order kinetics is the existence of a hydrogen threshold, below which the acetogen may only harvest a negligible amount of hydrogen from its environment. Both the previously presented models were insufficient to capture the dynamics of hydrogen uptake at concentrations approaching 100 mM, as seen in Fig. 1. Previous experimentation has found the threshold value for hydrogen uptake to be 1100 ± 200 ppm for *B. hydrogenotrophica* [14], which corresponds to 70 ± 12.7 mM.

There are several ways in which this threshold may be included in the model. The simplest method, variations of which have been applied in a number of comparable cases for other systems [23–26], is to formulate Eq. 1 as follows:

7 $$\frac{\mathrm{d}H}{\mathrm{d}t}=-\frac{{\mu }_{\mathrm{m}\mathrm{a}\mathrm{x}} X}{Y}\left(\frac{{H}^{\mathrm{*}}}{{K}_{H}+{H}^{\mathrm{*}}}\right),$$

where ${H}^{*}=H-{H}_{t}$, for hydrogen threshold concentration ${H}_{t}$. Equation 7 can be re-parameterised in the same manner as Eq. 4 to obtain:

8 $$\frac{\mathrm{d}H}{\mathrm{d}t}=-\frac{{\eta} {H}^{\mathrm{*}}X}{Y}.$$

Although simple and intuitive, this formulation is not robust for hydrogen concentrations below the threshold value, so is limited in its applications outside of simple monoculture cases. To prevent the model from predicting negative growth rates in such situations, the following condition was added:

$$H^{*} = 0\;{\text{when}}\;H < H_{t} .$$

This threshold modelling technique is referred to as T1.

An alternative model was proposed by Ribes et al. [27]. They construct the substrate equation as follows:

9 $$\frac{dH}{dt}=-\frac{{\mu }_{\mathrm{m}\mathrm{a}\mathrm{x}} X}{Y}\left(\frac{H-{H}_{t}f}{{K}_{H}+H-{H}_{t}f}F\right)$$

where $f$ and $F$ are both empirical sigmoidal functions that ensure growth decreases smoothly to 0 as the substrate concentration approaches the threshold value, ${H}_{t}$, and remains at 0 for concentrations below this threshold.

$$f=\frac{1}{1+\mathrm{exp}(A({H}_{t}-H))}$$

$$f=\frac{1}{1+\mathrm{exp}(A(T-H))}$$

This technique is referred to as T2. Equation 9 may be altered in the established manner to obtain:

10 $$\frac{\mathrm{d}H}{\mathrm{d}t}=-\frac{{\eta} XH}{Y}F.$$

$A$ and $T$ are tuning parameters of the model with limited biological significance, a discussion of which may be found in the original publication [27]. This approach is robust to all possible substrate concentrations, but adds considerable complexity and extra parameters to the model, which do not have a direct, biologically tangible meaning. This contrasts with the original Monod model (Eqs. 1–3), in which all parameters may be more easily interpreted and experimentally investigated.

The final technique outlined here is the use of reversible Michaelis–Menten kinetics [28]. This modelling technique is referred to as T3. It is assumed that the conversion of hydrogen and CO_2_ to acetate is a reversible reaction, with net conversion determined by the concentrations of each metabolite. The substrate equation is then:

11 $$\frac{\mathrm{d}H}{\mathrm{d}t}=-\frac{X}{Y}\left(\frac{{\mu }_{\mathrm{m}\mathrm{a}\mathrm{x},H}\frac{H}{{K}_{H}}-{\mu }_{\mathrm{m}\mathrm{a}\mathrm{x},R}\frac{P}{{K}_{R}}}{1+\frac{H}{{K}_{H}}+\frac{P}{{K}_{R}}}\right).$$

Here, ${\mu }_{\mathrm{m}\mathrm{a}\mathrm{x},H}$ and ${K}_{H}$ are the maximum rate and half-saturation constant for hydrogen consumption and ${\mu }_{\mathrm{m}\mathrm{a}\mathrm{x},R}$ and ${K}_{R}$ are the corresponding parameters for the reverse reaction. For this formulation, all the parameters have a direct interpretation in terms of the forward and reverse reactions. Note that we do not perform the simplification to first-order kinetics in this case, as Eq. 11 cannot be converted in the same manner as were Eqs. 7 and 9. Model T3 therefore consists of Eq. 11 in combination with Eqs. 5 and 6.

Importantly, the substitution of each of these techniques for a hydrogen threshold does not change the differential equations for product concentration or cell concentration.

As an extension to these model structures, we briefly consider the influence of a second substrate and metabolic pathway on the growth of *B. hydrogenotrophica*. This is used to analyse the effect of yeast extract on bacterial growth, performed in Fig. 5 and the “Discussion”. It is assumed that the bacterium feeds non-preferentially via both reductive acetogenesis and this second pathway. In both cases, acetate is the only product we consider. Let the concentration of yeast extract be denoted by $E$, so that, following first-order kinetics:

12 $$\frac{\mathrm{d}E}{\mathrm{d}t}=-\frac{{\eta}_{E} XE}{{Y}_{E}}.$$

The subscript $E$ denotes parameters that are specific to the metabolism of yeast extract. The inclusion of this equation in any of the previous models requires no change to the differential equation for hydrogen concentration, but does require additions to the differential equations for acetate concentration and bacterial growth, as follows:

13 $$\frac{\mathrm{d}P}{\mathrm{d}t}=-\frac{1}{4}\frac{\mathrm{d}H}{\mathrm{d}t}-{b}_{EP}\frac{\mathrm{d}E}{\mathrm{d}t}$$

14 $$\frac{\mathrm{d}X}{\mathrm{d}t}=-Y\frac{\mathrm{d}H}{\mathrm{d}t}-{Y}_{E}\frac{\mathrm{d}E}{\mathrm{d}t}-{k}_{d}X.$$

Here, ${b}_{EP}$ refers to the number of moles of acetate that are produced per mole of yeast extract consumed.

## Results

### Model calibration

**Fig. 2:**
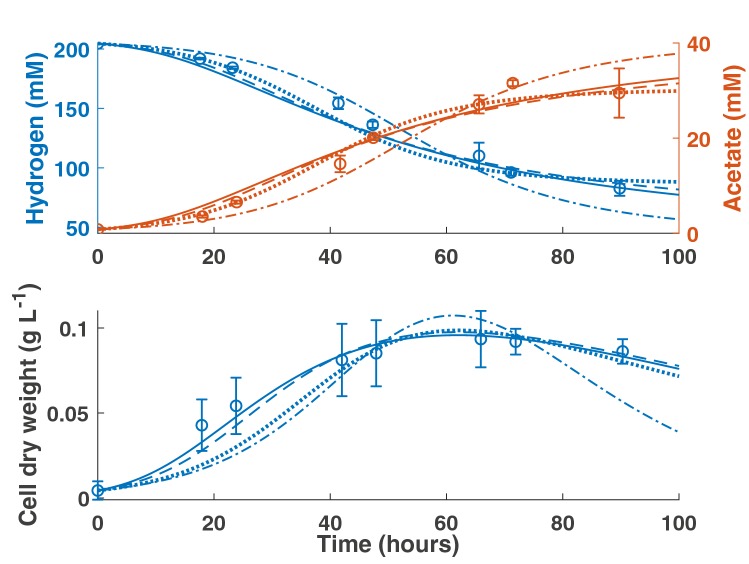
Model fits to data from Bernalier et al. (1996). Dash dot line: first-order kinetics model (Hydrogen *R*^2^ = 0.96; Acetate *R*^2^ = 0.94; CDW *R*^2^ = 0.76). Dotted line: T1 model (Hydrogen *R*^2^ = 0.97; Acetate *R*^2^ = 0.98; CDW *R*^2^ = 0.88). Dashed line: T2 model (Hydrogen *R*^2^ = 0.97; Acetate *R*^2^ = 0.96; CDW *R*^2^ = 0.95). Solid line: T3 model (Hydrogen *R*^2^ = 0.97; Acetate *R*^2^ = 0.96; CDW *R*^2^ = 0.98). Error bars denote standard deviations from three experimental determinations

We fitted the four model structures outlined in the methods to experimental data for *B. hydrogenotrophica* from Bernalier et al. [15]. Figure 2 shows a comparison of the model fits for the first-order kinetics model, T1, T2 and T3.

Model fitting of the T1 model gave an estimate of 86.2 mM (95% confidence interval 6.1–132.6 mM) for the threshold hydrogen concentration, which is comparable to the 70 ± 12.7 mM estimate of Leclerc et al. [14]. The best fits to the data were obtained using the T2 and T3 models, for which the *R*^2^ values for acetate, hydrogen and growth are all above 0.9. The parameter values obtained from model fitting are given in Table 1. The first-order kinetics model overpredicts the consumption of hydrogen at the final data point, as well as the corresponding data point for acetate production. In contrast, all three threshold models accurately predict the final data points for both metabolites, encouraging the use of a threshold model for this acetogen. The yield and cell death rate parameter values obtained from model fitting show good consistency between the threshold models.

**Table 1:** Best fit parameter values obtained from MCMC model fitting (95% confidence interval)

Parameter (units)	First-order kinetics	T1	T2	T3
$\eta$ (h^−1^ mM^−1^)	0.0008 (0.0007–0.0031)	0.0008 (0.0007–0.0032)	0.0054 (0.0018–0.0553)	–
$Y$ (g L^−1^ mM^−1^)	0.0037 (0.0026–0.0363)	0.0014 (0.0013–0.0192)	0.0017 (0.0012–0.0274)	0.0018 (0.0009–0.0072)
${k}_{d}$ (h^−1^)	0.087 (0.067–0.514)	0.014 (0.011–0.306)	0.019 (0.012–0.357)	0.022 (0.004–0.089)
${H}_{t}$ (mM)	–	86.2 (6.1–132.6)	–	–
$A$ (mM^−1^)	–	–	0.015 (0.0002–0.1096)	–
$T$ (mM)	–	–	336 (48–3509)	–
${\mu }_{\mathrm{m}\mathrm{a}\mathrm{x},H}$ (h^−1^)	–	–	–	0.451 (0.24–5.025)
${\mu }_{\mathrm{m}\mathrm{a}\mathrm{x},R}$ (h^−1^)	–	–	–	0.002 (0.001–0.058)
${K}_{H}$ (mM)	–	–	–	295.8 (55.3–395.8)
${K}_{R}$ (mM)	–	–	–	4.5 (0.14–6.4)

The non-linearized versions of models T1 and T2 were also investigated using the MCMC technique. However, as noted for the original Monod model, it was not possible to estimate the maximum growth rate and half-saturation constant reliably from the available data, resulting in estimates that were not biologically feasible. The non-linearized T1 model did show MCMC chain convergence for the threshold parameter (${H}_{t}$; Table 2), estimating its value at 83 mM (95% confidence interval 2.4–139.4 mM), comparable to the estimate of the T1 model. The non-linearized T2 model did not show MCMC chain convergence for the threshold parameter; therefore, the non-linearized model versions were not considered further.

**Table 2:** Mathematical notation

Notation	Description	Unit
$H$	Hydrogen concentration	mM
$P$	Acetate concentration	mM
$X$	Bacterial cell concentration	g L^−1^
$t$	Time	h
${\mu }_{\mathrm{m}\mathrm{a}\mathrm{x}}$ or ${\mu }_{\mathrm{m}\mathrm{a}\mathrm{x},H}$	Maximum growth rate on hydrogen	h^−1^
${\mu }_{max,R}$	Maximum rate of the reverse reaction	h^−1^
${K}_{H}$	Hydrogen half-saturation constant	mM
${K}_{R}$	Half-saturation constant for the reverse reaction	mM
$Y$	Yield of the bacterium when growing on hydrogen	g L^−1^ mM^−1^
${k}_{d}$	Bacterial death rate	h^−1^
$\eta$	First-order kinetics rate	h^−1^ mM^−1^
${H}_{t}$	Threshold concentration for hydrogen uptake	mM
${H}^{*}$	${H}^{*}=H-{H}_{t}$	mM
$f,F$	Sigmoidal smoothing functions from Ribes et al. [27]	Dimensionless
$A$	Tuning parameter from Ribes et al. [27]	mM^−1^
$T$	Tuning parameter from Ribes et al. [27]	mM

### Model validation

**Fig. 3:**
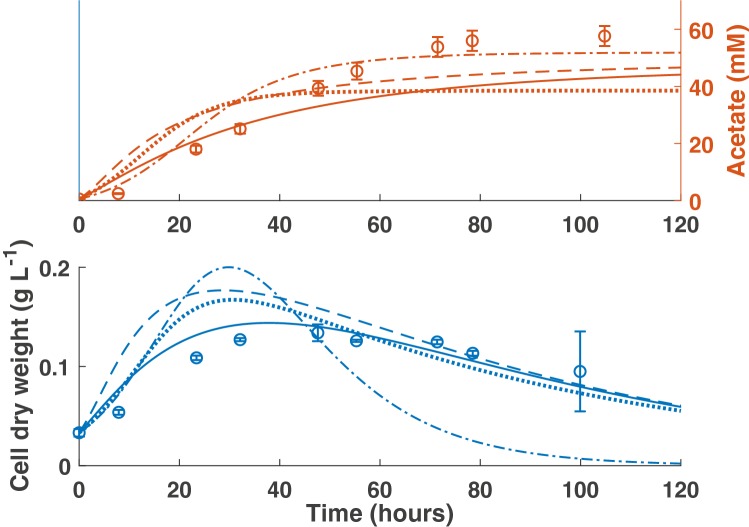
Model validation against data from Groher, Weuster–Botz [19] in which *B. hydrogenotrophica* was grown on GA medium. Dash dot line: first-order kinetics model (Acetate *R*^2^ = 0.91; CDW *R*^2^ = 0.15). Dotted line: T1 model (Acetate *R*^2^ = 0.31; CDW *R*^2^ = 0.66). Dashed line: T2 model (Acetate *R*^2^ = 0.63; CDW *R*^2^ = 0.51). Solid line: T3 model (Acetate *R*^2^ = 0.58; CDW *R*^2^ = 0.84). Error bars denote standard deviations of at least three experimental replicates

To validate the threshold models, we compared the predictions from each model with separate experimental data from Groher, Weuster–Botz [19]. Two sets of time-course data were obtained for *B. hydrogenotrophica*, one set from the bacterium grown on the newly proposed general acetogen medium (GA) and a second set using the recommended DSMZ medium. A comparison of the predictions of the first-order kinetics model and the three threshold models with the GA medium data is shown in Fig. 3.

There is clear variation between the predictions of each of the four model structures. The first-order kinetics model was the most accurate in predicting acetate production, but the least accurate in predicting bacterial growth. The converse is true of T3, the reversible Michaelis–Menten kinetics model, which predicted bacterial growth accurately, but underestimated acetate production.

**Fig. 4:**
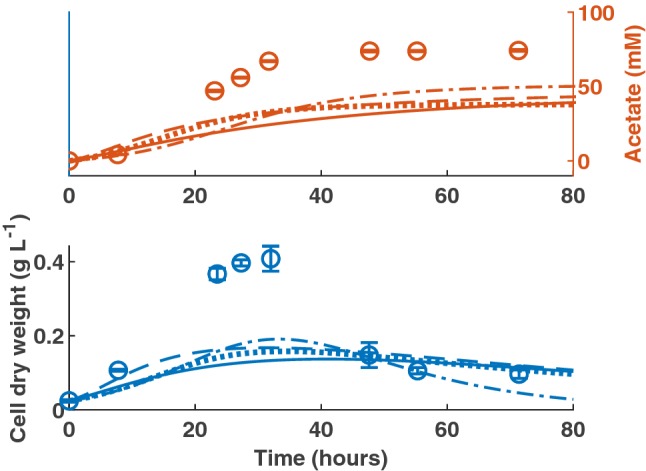
Model validation against data from Groher, Weuster–Botz [19] in which *B. hydrogenotrophica* was grown on DSMZ medium. Dash dot line: first-order kinetics model. Dotted line: T1 model. Dashed line: T2 model. Solid line: T3 model. All *R*^2^ values were < 0.20. Error bars denote standard deviations of at least three experimental replicates

Figure 4 shows the predictions of each of the four models against the DSMZ medium data. None of the models perform well in this scenario, but this may be attributed to the wide differences between the DSMZ, GA and calibration data media, which are explained in “Discussion”.

## Discussion

We may use our models to draw several conclusions on both the metabolism of *B. hydrogenotrophica* and the mathematics of the models themselves. The results of the calibration encourage the inclusion of threshold considerations in mathematical models for reductive acetogens. The threshold models presented here captured the monoculture growth kinetics of the acetogen more accurately than the first-order kinetics model. This is to be expected, given that *B. hydrogenotrophica* has previously been shown to have an uptake threshold for hydrogen of approximately 70 mM [14], which was supported by our estimate of 86.2 mM using model T1. However, deciding which of the three threshold models is best suited to modelling the growth of this acetogen is challenging. Each performed similarly when compared to the calibration data, but significant variation was seen in the predictions for the validation data. Although the first-order kinetics model gave a prediction of acetate production with *R*^2^ > 0.9, this model structure was unable to capture the growth data. The T1 model also yielded predictions for both metabolite concentrations and growth that were unsatisfactory for the validation data.

Between the T2 and T3 model structures, there is no clearly superior outcome in terms of model fit. Both require a greater number of parameters than the other models presented (two or three more parameters than the first-order kinetics model for the T2 or T3 models respectively), some of which cannot be easily estimated experimentally. With fewer parameters, the T2 model is the simpler of the two, but the T3 model features parameters that are more biologically tangible. While the T3 model was more accurate in predicting CDW, it underpredicted acetate production for the validation dataset. Given further validation data from batch experiments, it may be possible to determine which of the threshold models is most effective, but this data is currently unavailable.

It should also be noted that the T3 model is not well suited to modelling environments with high acetate concentrations and low hydrogen concentrations. Using the parameter values from Table 1, the non-trivial steady state for metabolite concentrations is achieved when $P\approx 3.43 H$. This is an attraction point, stable to small perturbations in the variables. Therefore, in scenarios with a hydrogen concentration lower than the steady state value, the model would predict depletion of acetate and accumulation of hydrogen until the steady state was reached. We would also expect any bacterial growth yield to be different if performing the reverse reaction, if indeed this reaction could be performed by the strain in question. The current model structure would predict a reduction in biomass in this situation, so would need to be altered with an appropriate yield value included. As we are not aware of any data for the culture of reductive acetogens under such conditions for comparison, we would not recommend the use of the T3 model in this scenario. Moreover, the fact that the consumption of hydrogen is limited by the acetate concentration rather than low concentrations of hydrogen is not consistent with the results of Leclerc et al. [14] for a hydrogen threshold. We therefore believe that the T2 model with the threshold term defined by Ribes et al. [27] is the most appropriate for the data considered here.

Ribes et al. [27] suggest the following values for the T2 parameters $A$ and $T$:

$$A=\frac{100}{{H}_{t}},$$

$$T=1.1{H}_{t}.$$

While these values give some biological meaning to the parameters by relating them to the substrate threshold for the bacterium, they are not appropriate in all cases. Using the threshold hydrogen value of 86.2 mM, this would give values of $A\approx 1.16$ and $T=94.82$, each of which are 773% and 30% the value of the T2 estimates for these parameters, respectively. Use of these values in the model results in an abrupt inhibition of hydrogen metabolism and growth with poor model fit (data not shown), so is not appropriate for modelling the hydrogen threshold of *B. hydrogenotrophica*. $A$ and $T$ should be seen as related to ${H}_{t}$, and thus biologically tangible in this sense; however, the exact relationship between these parameters will likely be case dependent.

There have been previous models of *B. hydrogenotrophica* growth and metabolism. Tamayo et al. [29] employed a Monod-based model including gas–liquid transfer and the consideration of both viable and dead cells, but did not include a threshold hydrogen concentration. Their model structure and the units used meant that a direct comparison to each of our parameter values was impossible, but the death rates they obtained (mean rate: 0.03 h^−1^) are similar to those found here. More recently, D'Hoe et al. [30] formulated a mathematical model for *B. hydrogenotrophica* for use in combination with models for two other GIT bacteria. Their model was based on experimental data presented in the same publication, but focusses on the ability of this acetogen to feed on formate and fructose, omitting its ability to consume CO_2_ and H_2_. The model was constructed in this manner as no detectable consumption of either metabolite was found in culture, which the authors assume is because these metabolites did not reach sufficient concentrations for *B. hydrogenotrophica* to metabolise. Indeed, the concentration of hydrogen remained below 40 mM in monoculture and does not appear to have increased above 70 mM, the hydrogen threshold proposed by Leclerc et al. [14] for this bacterium, in any of the co-culture combinations studied. It is unclear whether this is due to depletion of formate and fructose in the medium, from which hydrogen was being produced, or whether hydrogen was metabolised by *B. hydrogenotrophica* at concentrations approaching the threshold value, thus maintaining the low concentration. It is also unclear whether *B. hydrogenotrophica* preferentially metabolises formate and fructose over hydrogen at these concentrations. However, even without consideration of hydrogen metabolism, the authors obtained a good model fit to their data. Any complete future model for the acetogens must include the metabolism of all possible substrates, to be applicable to complex multi-substrate and multi-product environments such as the GIT or a bioreactor.

The medium in which the bacteria are grown will have an enormous effect on experimental results and, therefore, the quality of the model prediction. The purpose of Groher, Weuster-Botz [19] was to present a new medium better suited to the monoculture growth of acetogens and acetate production. Indeed, they found that *B. hydrogenotrophica* was more efficient in terms of cell specific acetate formation on the GA medium than on the DSMZ recommended medium. It is notable that the acetogen achieved more rapid growth and to a greater concentration on the DSMZ medium, but the authors state that this is almost certainly due to the greater concentration of complex constituents in the DSMZ medium compared to the GA medium. Critically, the DSMZ medium contains 25 g L^−1^ yeast extract, compared to 2 g L^−1^ in the GA medium. The medium used by Bernalier et al. [15] contained 0.5 g L^−1^ yeast extract, making it more comparable in this respect to the GA medium than the DSMZ medium. We therefore expect our models to be more accurate in predicting growth on the GA medium, than a medium that contains significantly more complex compounds. This justifies the lack of fit displayed in Fig. 4. Previous experimental work with this bacterium has found that the production of acetate is limited by the concentration of yeast extract in the medium, but only concentrations up to 4 g L^−1^ were considered [31]. The additional carbon source provided by the yeast extract likely results in significant bacterial feeding via pathways other than reductive acetogenesis, for which our original threshold models were not designed to account.

This is supported by considering the stoichiometry of reductive acetogenesis given the available hydrogen. At the beginning of both the GA and DSMZ experiments, the 400 mL gaseous headspace in the serum bottles used was occupied by a gas mixture of H_2_:CO_2_ (66:34) at 200 kPa pressure. This 0.02 mol of hydrogen could theoretically produce at most 50 mM of acetate in the 100 mL medium via the Wood–Ljungdahl pathway. This is approximately equal to the acetate yield in the GA medium, but is around 20 mM lower than the acetate yield in the DSMZ medium. This confirms that a significant proportion of the acetate produced on the DSMZ medium in this experiment was not derived from reductive acetogenesis, and therefore must be the result of alternative carbon source consumption.

**Fig. 5:**
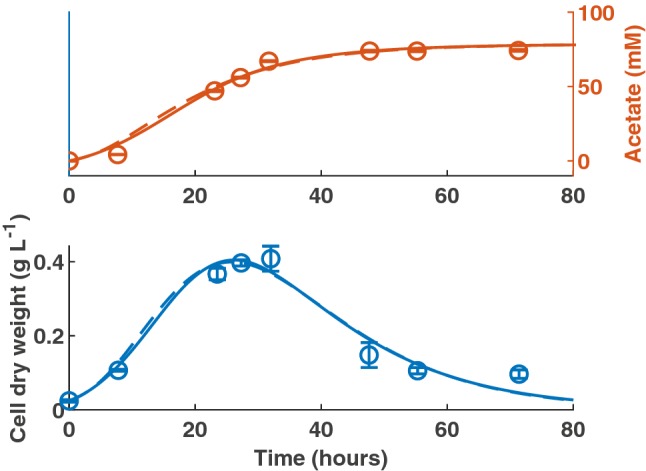
Example model prediction for *B. hydrogenotrophica* growth on DSMZ medium with the inclusion of yeast extract metabolism. Dashed line: T2 model (Acetate *R*^2^ = 0.98; CDW *R*^2^ = 0.96). Solid line: T3 model (Acetate *R*^2^ = 0.99; CDW *R*^2^ = 0.96). Error bars denote standard deviations of at least three experimental replicates. Parameter values used during yeast extract metabolism simulation were: ${\eta}_{E}=0.0018$ h^−1^ mM^−1^, ${Y}_{E}=0.02$ g L^−1^ mM^−1^, ${b}_{EP}=0.43$ and ${k}_{d}=0.112$ h^−1^ for T2; ${\eta}_{E}=0.002$ h^−1^ mM^−1^, ${Y}_{E}=0.013$ g L^−1^ mM^−1^, ${b}_{EP}=0.5$ and ${k}_{d}=0.08$ h^−1^ for T3

The T2 and T3 models were altered by including Eqs. 12–14 to ascertain whether growth on constituents of the added yeast extract may be responsible for increased growth and acetate yield in the DSMZ medium. As there is no available time-course data for the growth of this bacterium on yeast extract, the parameter values specific to yeast extract metabolism were drawn from model fitting to the *B. hydrogenotrophica* growth on DSMZ medium data. As shown in Fig. 5, the inclusion of yeast extract metabolism allows both the T2 and T3 models to capture growth and acetate production on DSMZ medium.

We cannot draw conclusions about the growth of *B. hydrogenotrophica* on yeast extract from these data, since yeast extract concentration was not measured over time, nor were metabolites other than acetate. This leaves us uncertain of the proportion of carbon from the yeast extract that was converted to compounds other than acetate. Therefore, the parameter values for ${\eta}_{E}$, ${Y}_{E}$, ${b}_{EP}$ and ${k}_{d}$ used to generate Fig. 5 and stated in the caption are entirely hypothetical and should not be treated as accurate estimates of these parameter values. More experimental work is required to understand yeast extract metabolism by this bacterium, but Fig. 5 does demonstrate that it is possible to account for the increased production of acetate, as well as the bacterial cell concentration, by including an additional carbon source in the model.

The results of our models support the assertion of Groher, Weuster–Botz [19] that their GA medium allows for culture of acetogens with less confounding factors, further recommending its use for assessment of industrial suitability of acetogenic strains. However, while our models are sufficient for the study of reductive acetogenesis under batch conditions, it is important to note that in many environments, acetogens such as *B. hydrogenotrophica* have the ability to use a number of metabolic pathways and substrates [16, 30]. Such dynamics must be included in any mathematical model that wishes to fully capture the growth and metabolism of these bacteria in more complex scenarios.

One such scenario where a modelling perspective would be useful, is in the competition for hydrogen between acetogens and other hydrogenotrophs. It is thought that both sulphate-reducing bacteria and methanogens should outcompete acetogens for hydrogen due to their more efficient metabolism of this substrate [6]. However, in environments such as the human GIT, acetogens are observed to coexist with the other hydrogenotrophs [32]. While much study has been devoted to this topic, the key factors behind GIT hydrogenotroph ecology remain unclear [3]. Threshold models such as those presented here could be applied to the competition between the three hydrogenotroph types and may provide more insight into their coexistence in the GIT.

In conclusion, we have found that the use of threshold models is a more effective way to capture acetogen growth and metabolic dynamics than the simple Monod model, although finding which threshold model is most effective requires further investigation and further experimental data. Our models and analyses provide further evidence for minimal acetogen growth below a threshold hydrogen concentration. Moreover, using our models, we found evidence to support the assertion of Groher, Weuster-Botz [19] that their GA medium is superior to the DSMZ medium in terms of growth assessment and, in the case of *B. hydrogenotrophica*, cell specific acetate production, due to the reduced concentration of complex medium constituents. The differences in the results between different media discussed here will be broadly applicable to the culturing of many microorganisms and models such as these can be useful tools in investigating such discrepancies.
